# Talking about diseases; developing a model of patient and public-prioritised disease phenotypes

**DOI:** 10.1038/s41746-024-01257-8

**Published:** 2024-09-30

**Authors:** Karin Slater, Paul N. Schofield, James Wright, Paul Clift, Anushka Irani, William Bradlow, Furqan Aziz, Georgios V. Gkoutos

**Affiliations:** 1 Institute of Cancer and Genomic Sciences, University of Birmingham, Birmingham, UK; 2 Centre for Environmental Research and Justice, University of Birmingham, Birmingham, UK; 3 Centre for Health Data Science, University of Birmingham, Birmingham, UK; 4 University Hospitals Birmingham NHS Foundation Trust, Birmingham, UK; 5 Department of Physiology, Development, and Neuroscience, University of Cambridge, Cambridge, UK; 6 White Swan Charity, London, UK; 7 Nuffield Department of Clinical Neurosciences, University of Oxford, Oxford, UK; 8 Division of Rheumatology, Mayo Clinic Florida, Jacksonville, FL USA; 9 School of Computing and Mathematical Sciences, University of Leicester, Leicester, UK

**Keywords:** Diseases, Health care economics, Signs and symptoms

## Abstract

Deep phenotyping describes the use of standardised terminologies to create comprehensive phenotypic descriptions of biomedical phenomena. These characterisations facilitate secondary analysis, evidence synthesis, and practitioner awareness, thereby guiding patient care. The vast majority of this knowledge is derived from sources that describe an academic understanding of disease, including academic literature and experimental databases. Previous work indicates a gulf between the priorities, perspectives, and perceptions held by different healthcare stakeholders. Using social media data, we develop a phenotype model that represents a public perspective on disease and compare this with a model derived from a combination of existing academic phenotype databases. We identified 52,198 positive disease-phenotype associations from social media across 311 diseases. We further identified 24,618 novel phenotype associations not shared by the biomedical and literature-derived phenotype model across 304 diseases, of which we considered 14,531 significant. Manifestations of disease affecting quality of life, and concerning endocrine, digestive, and reproductive diseases were over-represented in the social media phenotype model. An expert clinical review found that social media-derived associations were considered similarly well-established to those derived from literature, and were seen significantly more in patient clinical encounters. The phenotype model recovered from social media presents a significantly different perspective than existing resources derived from biomedical databases and literature, providing a large number of associations novel to the latter dataset. We propose that the integration and interrogation of these public perspectives on the disease can inform clinical awareness, improve secondary analysis, and bridge understanding and priorities across healthcare stakeholders.

## Introduction

Deep phenotypes have the power and potential to support unprecedented advances in healthcare^1^. While deep phenotyping is most often employed to construct phenotypic profiles of patients to facilitate personalised and precision medicine, for example, in patient population stratification and diagnosis^2,3^, they may also be used to operationally define diseases themselves.

Organised deep phenotypes for diseases were historically developed for databases of rare disease phenotypes manually curated from patient descriptions^4,5^. With the advent of more informatics-led approaches, these resources were extended to describe common, complex, and rare diseases, with associations between diseases and phenotypes being identified through analysis of data derived from experiments, manual curation, and information extraction from the literature. For example, Hoehndorf et al.^6^ explored the human diseasome in the context of variant prioritisation and thematic analysis of inter-related disease areas^7^, while Kafkas et al.^8^ linked literature-mined phenotype data to BioBank profiles and another recent literature-based approach performed by Pilehvar et al.^9^, integrating modern natural language processing approaches. These resources provide a rich source of background knowledge, which has proven useful for a wide range of tasks, including differential diagnosis^10,11^ and causative genetic variant prediction^12,13^. They also provide a data-driven method for the explication of diseases, which can be interrogated for supporting clinical awareness of disease symptoms^14^ and identifying sub-types in the case of diseases with complex or spectral presentation^15^, thereby supporting and facilitating healthcare across multiple stages of translation and implementation.

Nevertheless, the more recent drive for digital phenotyping^1^, a sub-field of deep phenotyping that aims to integrate novel data sources, such as wearable devices and social media, into our understanding of disease, has largely not found its way to contributing to those background knowledge resources describing diseases themselves, despite being increasingly popular in patient-level investigations^1^. As such, the background knowledge resources continue to be derived almost exclusively from institutional sources of knowledge, largely academic literature and experimental data. Scientific knowledge resources reflect scientific interest, which is influenced by cultural trends, funding availability, and personal interest^16^. This leads to an imbalance in the development of knowledge, with scientific attention not always being fully aligned with need or equity. These scientific knowledge resources form the substrate from which treatment guidelines are synthesised and, thereby, from which medicine is practised.

Similar imbalances occur in direct healthcare practice, where social biases play out in medical training and practice, generating disadvantages for sub-populations, for example, on the basis of gender, race, age, geography, and economic class. Many such groups have characteristic health problems which are consequently addressed poorly, or not at all. For example, the tendency for medical professionals to ignore or downplay reports of symptoms by women is widely reported and explored in literature^17^. These issues are associated with reduced quality of care, extremely long delays in the diagnosis of diseases, for example, endometriosis^18^, or causing a large proportion of cardiologists self-reporting as unprepared to diagnose cardiovascular disease in women^19^. In a related example, exposure to topics of transgender health is extremely low in medical curricula^20^; this is then manifest in the experience of those individuals facing high levels of discrimination seeking healthcare^21^ and in poor routine data collection, negatively affecting resources that are used for medical research^22^. These issues are compounded by the inherent limitations in statistical power when considering minority cohorts^23^. In some cases, these issues translate to a more limited understanding of the diseases from which these groups are more likely to suffer^24^. A recent study reports a strong preference amongst clinicians for downgrading the importance of patient-reported symptoms, misattributing them either through misunderstanding of terms used or pre-judgement, particularly with constitutional symptoms^25^, emphasising the existence of an underlying semantic mismatch between patient and health professional discourse.

The combination of such limitations in scientific exploration and medical care is deeply inter-linked and self-perpetuating. If an incomplete understanding of a disease informs experimental design, clinical care, and clinical data collection, these data and outcomes are then used to inform new academic research, and, in turn, the clinical practice informed by it. Fundamentally, this feedback loop limits the ability to discover and integrate new perspectives that could lead to improved understanding and treatment of diseases. In addition, previous research has shown that patients and clinicians hold different perspectives and priorities concerning disease^26,27^. For example, patients usually consider news of a ‘stable’ cancer as being negative, while doctors often consider this to be positive^28^. Meanwhile, analysis of an online Uveitis patient forum identified different language and priorities than those expressed by existing scientific and clinical knowledge resources^29^.

To bridge these gaps in understanding diseases, the patient-centred care paradigm aims to integrate the perspectives and priorities of patients and the public into clinical care^30^. Patient reported outcome measures and quality-of-life assessments are methods used to identify patient perspectives and to reconcile them with clinical understanding. However, patient and public-centred approaches to developing knowledge resources and phenotype models for clinical practice and research have been under-explored. Lenzi et al.^31^ used a topic modelling approach to discern a digital phenotype for diabetes based on an Italian patient forum. Meanwhile, Maggio et al.^32^ focused on the discussion of methodological aspects of digital phenotyping from social data, and linked data retrieved from social media to epidemiological data. However, existing approaches focus on single diseases, do not correlate their knowledge with literature resources in a systematic way, provide a framework for hypothesis generation for novel associations, and do not provide an open database for secondary research use. Meanwhile, the approaches that focus on creating phenotypes for large numbers of diseases, in addition to being derived entirely from literature and biomedical resources, are rarely actively evaluated in a real clinical context.

In this work, we propose an approach to developing a phenotype model to represent and expose patient and public perspectives and knowledge on disease. We develop a social media phenotype model that identifies disease-phenotype associations for a range of common and rare diseases. We hypothesise that the perspectives presented by this resource will significantly differ from existing knowledge resources. To test this hypothesis, we create a consolidated Biomedical Database and Literature Phenotype with which to compare and contrast the social media-derived associations, highlighting novel associations and differences in theme. We then perform a clinical evaluation of associations for several diseases, including an evaluation of the feasibility of novel phenotype associations found in public discourse but currently unknown to, or underappreciated in the literature and clinical practice.

## Results

We analysed 68,319,325 records of social media posts that describe mentions of a set of 488 diseases, annotated to Disease Ontology (DO)^33,34^ classes based on mentions of their labels in a social media text. Table 1 shows that the posts were sourced from a range of social media, mostly comprised of Twitter, Reddit, with smaller contributions from online fora and review narratives. A total of 5895 unique phenotypes, linked to the Human Phenotype Ontology (HPO)^35^ were mentioned across all posts. From these data, we developed the Social Media Phenotype (SMP), which describes 52,198 positive associations for 311 diseases, with 620 unique phenotypes appearing across all associations. Of those, we consider 35,381 to be significantly associated, using an acceptable false discovery rate of 0.0005. We also identified a Biomedical Database and Literature Phenotype (BDLP) by combining disease-phenotype associations defined by multiple works from a combination of structured databases and literature-based text analysis. To facilitate a comparative analysis of these phenotype models, we linked profiles for 304 diseases from the SMP to their equivalents in the BDLP.

**Table 1:** Document count and contribution by source

Source	Document count	Contribution to total (%)
Twitter	51,621,597	76
Reddit	13,712,022	20
Boards (online forums)	2,607,165	4
Reviews	378,541	1

We created an online resource for exploring and comparing the SMP and BDLP, which is freely available at https://phenotype.digital/. The produced phenotype models are also made freely available^36^.

### Comparative analysis of SMP and BDLP

Across the 304 linked diseases, social media phenotype profiles strongly recapitulated the BDLP, with a disease reclassification analysis revealing an AUC of 0.872 (0.95 = 0.855–0.889). The SMP describes 24,618 novel phenotype associations: those not appearing in the disease-matched profile in the BDLP—either explicitly or in a more specific form. Conversely, the BDLP contained 186,144 phenotypes not recovered in the SMP, of a total of 204,158 associations. Of the 24,618 novel phenotype associations in the SMP, 14,531 were considered statistically significant.

The DO and HPO organise diseases and phenotypes into a hierarchical classification structure. For example, lower back pain and back pain can both be considered kinds of pain. This allows us to organise the diseases and phenotypes into groups using inference based on ontology. DO was selected from the alternatives as the disease framework because its structure contains clinically and biologically meaningful axes of classification. Table 2 lists the ten most common groups of phenotypes appearing in novel associations, excluding very general phenotypes. Pain was the most frequently appearing group, with 6% of all novel phenotype associations being in this group.

**Table 2:** Top ten most frequently associated groups of novel phenotypes in the SMP

Phenotype	Inclusion
Pain (HP:0012531)	0.06
Functional abnormality of the gastrointestinal tract (HP:0012719)	0.05
Abdominal symptom (HP:0011458)	0.03
Abnormality of reproductive system physiology (HP:0000080)	0.03
Abnormality of blood circulation (HP:0011028)	0.03
Internal haemorrhage (HP:0011029)	0.03
Abnormal cell morphology (HP:0025461)	0.02
Abnormal emotion/affect behaviour (HP:0100851)	0.02
Vascular skin abnormality (HP:0011276)	0.02
Abnormal myeloid cell morphology (HP:0020047)	0.02

Proportion of novel phenotypes refers to the proportion of phenotypes in the SMP dataset that were not included in the BDLP for the 304 matched diseases. For example, 6% of all novel phenotype associations were either pain or more specific kinds of pain (e.g. abdominal pain). Groups of phenotypes with low information content below 0.6 Resnik, were excluded.

The hierarchical structure of the ontologies also allows us to explore the associations according to the very general groupings of diseases and phenotypes that sit at the top of these structures, such as respiratory phenotypes or metabolic diseases. Figure 2 shows the representation of phenotype associations according to those categories in the HPO and DO. Figure 2a shows a comparison of associations for SMP and BDLP falling under each category in HPO, with constitutional symptoms, as defined by HPO, being clearly over-represented relative both to other facets in the SMP and to all facets in the BDLP. Other far smaller differences between the two sets are revealed in a greater expression of voice, thoracic cavity, and blood phenotypes in the BDLP, and a greater proportion of growth anomaly and digestive phenotypes appearing in the SMP. These proportions are also reflected across the novel subset of the SMP, shown in Fig. 2b, which shows a largely similar distribution of facet expression to that of the full SMP. Figure 2a and b show that the distribution of novel phenotypes in SMP is very similar to the overall distribution of phenotypes in BLP. Figure 2c shows the the proportion of novel phenotypes in the SMP accorded to each disease category defined by the DO, projected onto the distribution of the membership of the total 304 diseases, showing that these are strongly correlated, though with a greater focus on digestive diseases, and a lesser focus on infectious and mental health diseases.

**Fig. 1:**
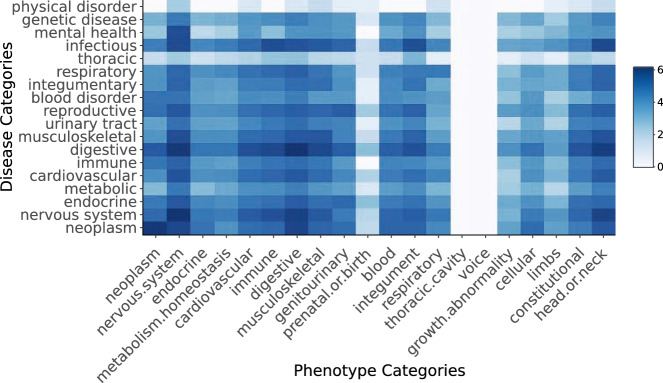
Heatmap of disease and phenotype categories for novel and significant social media phenotype associations. The heatmap displays the normal log occurrence of novel and significant associations across disease and phenotype categories in the 304 matched SMP diseases. The 14 pairs of disease and phenotype categories that refer to matching biological systems are placed symmetrically and lead the rows and columns.

Figure 1 shows the correlative proportions of significant novel phenotypes for disease and phenotype categories. Of these, 14 were able to be linked by shared anatomical systems, such as ‘nervous system phenotypes’ and ‘nervous system diseases.’ Some of the most strongly represented pairs were matching, such as digestive, with a log occurrence of 6.17, the nervous system with 6.15, and neoplasm with 6.07. The heatmap also shows a paucity of novel associations for thoracic cavity and voice across all disease categories, though thoracic diseases show some positive relationship with integumental phenotypes. Novel associations for physical disorders were mostly concentrated in nervous system phenotypes, with some contribution also from the head or neck.

**Fig. 2:**
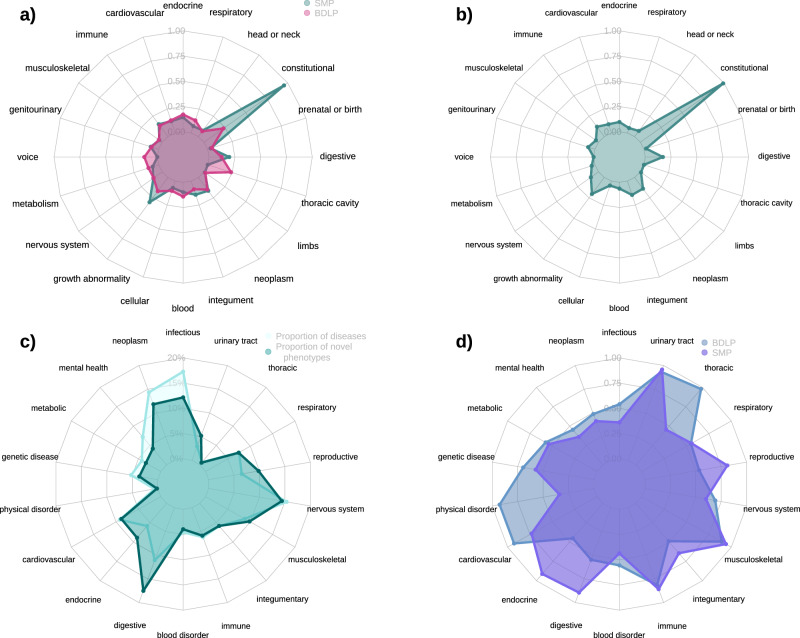
Thematic analyses of phenotype associations in the BDLP and SMP according to high-level organisational categories. Categories are defined by high-level classes in HPO and the DO, with membership being accorded based on the transitive sub-class relation. Phenotype and disease membership in categories are not mutually exclusive: for example, `lung cancer' may be considered both a neoplasm and a respiratory disease. **a** Differential expression of phenotype associations in the Biomedical Database and Literature Phenotype and the Social Media Phenotype across high-level categories in the Human Phenotype Ontology, proportional to the overall number of phenotypes of that category defined in the ontology. **b** Distribution of novel and significant phenotype associations in the Social Media Phenotype across high-level categories in the Human Phenotype Ontology, proportional to the overall number of phenotypes of that category defined in the ontology. The distribution of novel phenotype categories is observably very similar to the overall phenotypes, observable in (**a**). **c** Proportion of novel and significant phenotypes assigned to each disease area defined by the DO. `Proportion of diseases' refers to the proportion of the 304 diseases for which the SMP contains associations that fall under each disease area. The proportion of novel associations belonging to each category is strongly correlated with the proportion of total diseases in that category (Spearman rho = 0.889; *p* = 7.634e−07). **d** Proportion of constitutional symptom phenotype associations defined by BDLP and SMP belonging to each disease category, proportional to the total diseases in each category. Proportions were not significantly correlated (Spearman rho = 0.189; *p* = 0.451).

**Fig. 3:**
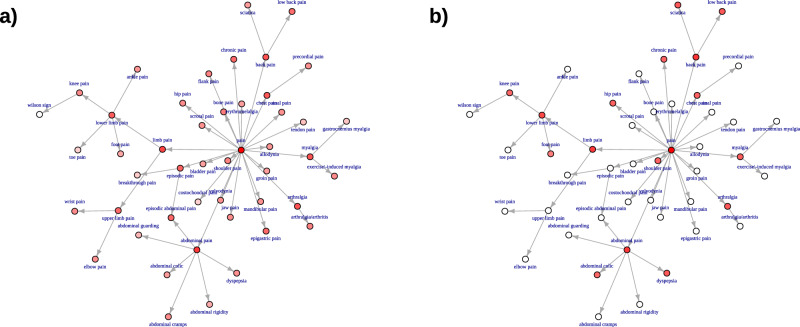
Network projection of pain phenotype associations for the BDLP and SMP. The graph indicates fewer phenotypes represented in the SMP, with a stronger focus on more general, or high-level, phenotypes that are more closely linked to pain, with associations being more widely distributed across the full set of nodes in the BDLP. **a** Pain BDLP. **b** Pain SMP.

Due to its clear over-representation in the SMP dataset, we further investigated associations that fell under the constitutional symptoms facet. There are 3693 constitutional symptom associations in the BDLP, and 2289 in the SMP, with 1264 of those being novel (not included in the BDLP). Figure 2d shows the differential distribution of constitutional phenotype associations for the BDLP and SMP across disease categories, proportional to the total number of the linked diseases in each category. This shows that there is a greater focus on constitutional symptoms in the BDLP for thoracic, genetic, cardiovascular, physical, and, to a lesser extent, nervous system, disorders. Meanwhile, the SMP exhibits a greater focus on constitutional symptoms in reproductive, endocrine, digestive, and integumentary diseases. Across all constitutional symptom associations in both sets, there are only 86 unique classes, with a total of only 28 unique classes appearing across the SMP, with all 86 appearing in the BDLP. Table 3 shows the top groups of associated phenotypes in the BDLP and SMP. In both phenotype models, pain is by far the most compositional constitutional symptom group (BDLP 68%, SMP 74%), with other contributions from impairment of activities of daily living, impaired continence, and dyspepsia. The distribution of phenotypes is mostly similar across the two sets of phenotypes, with notable increases in the proportion of back pain (+10%), lower limb pain (+4%) and sciatica (+4%) in the SMP. Conversely, the BDLP contains notably greater proportions of chills (+4%). Figure 3 shows a projection of pain phenotype associations for BDLP and SMP, showing clearly that in the SMP, phenotype associations are far more strongly concentrated on a smaller number of phenotypes, with associations being more distributed across the entire sub-graph in the case of BDLP.

**Table 3:** Top constitutional phenotypes found in the BDLP and SMP

Class	Proportion of associations	
	BDLP	SMP
Pain (HP:0012531)	0.68	0.74
Abdominal pain (HP:0002027)	0.11	0.12
Limb pain (HP:0009763)	0.08	0.13
Fatigue (HP:0012378)	0.07	0.05
Impairment of activities of daily living (HP:0031058)	0.06	0.05
Arthralgia (HP:0002829)	0.05	0.05
Back pain (HP:0003418)	0.05	0.15
Myalgia (HP:0003326)	0.05	0.04
Impaired continence (HP:0031064)	0.05	0.05
Chest pain (HP:0100749)	0.04	0.03
Chills (HP:0025143)	0.04	0
Lower limb pain (HP:0012514)	0.04	0.08
Pain in head and neck region (HP:0046506)	0.04	0.04
Night sweats (HP:0030166)	0.02	0.04
Dyspepsia (HP:0410281)	0.01	0.04
Urinary incontinence (HP:0000020)	0.02	0.05
Low back pain (HP:0003419)	0.02	0.05
Sciatica (HP:0011868)	0.01	0.05
Neck pain (HP:0030833)	0.01	0.04
Chronic pain (HP:0012532)	0.03	0.04
Halitosis (HP:0100812)	0.01	0.04

Proportion of associations is the percentage of constitutional symptom phenotype associations that were equivalent to or more specific than the named phenotype. Criteria for inclusion was at least 0.04 proportion of constitutional symptoms in either SMP or BDLP.

### Clinical review and hypothesis generation

**Fig. 4:**
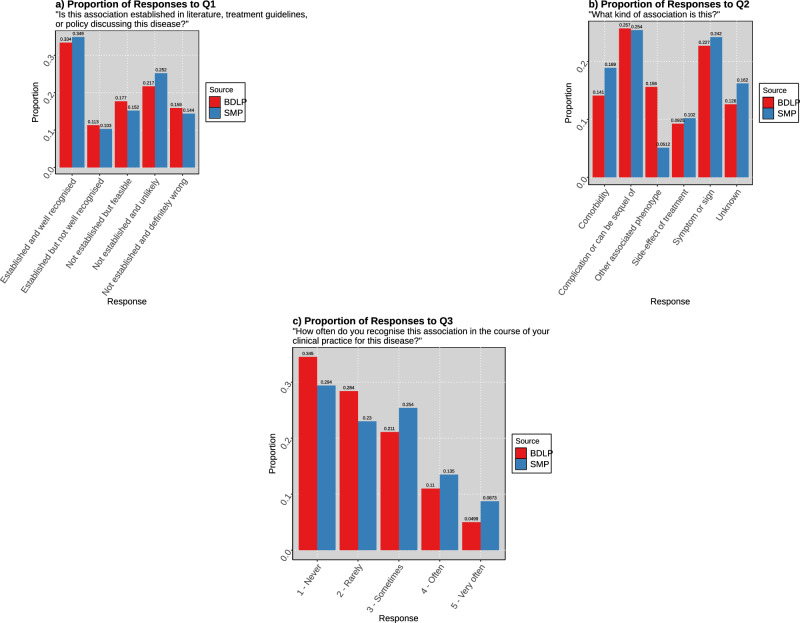
Responses to clinical review questions for disease-phenotype associations. These are for the 10 diseases marked by the clinical reviewers, separated by their membership in SMP and BDLP. If an association was recovered by both databases, it is included in both sets here. Reviewers were blinded to the source of the phenotype association. **a** Proportion of clinician responses to the question “Is this association established in literature, treatment guidelines, or policy discussing this disease?” across the set of ten disease responses. **b** Proportion of clinician responses to the question “What kind of association is this?” across the set of ten disease responses. **c** Proportion of clinician responses to the question “How often do you recognise this association in the course of your clinical practice for this disease?” across the set of ten disease responses.

Twelve clinical reviewers evaluated phenotype associations for 13 diseases, in total returning 13,088 question responses. The first question evaluated how valid they thought the association was (Fig. 4a), with strongly correlated distributions (*X*^2^ = 47.504, *p* = 1.198e−09), with a slightly greater proportion of ‘not established and unlikely’ results. The second question concerned the type of phenotype association (Fig. 4b), and the distribution of responses was also strongly correlated (X^2^ = 76.435, *p*-value = 4.667e−15). However, this question contains very different response proportions for certain categories. ‘Other associated phenotype,’ was much more common for BDLP associations (0.156) than for those in the SMP (0.052), while ‘Comorbidity,’ and ‘Unknown,’ were seen far more often in response to SMP associations. The third question queried how often the clinicians saw patients with the phenotypes. SMP phenotypes were seen more often in the clinic than those in the BDLP (one-tailed Wilcoxon rank-sum test p = 3.868e−05). Despite this trend, both the SMP and BDLP were heavily skewed towards infrequently observed phenotype associations, with more than half of all associations being observed ‘never’ or ‘rarely’ in both cases. *p*-values remain relevant with Bonferroni correction.

Using the results of the clinical review, we were also able to shortlist sets of phenotypes that are potentially novel. To do this, we isolated responses that were considered ‘Not established but feasible,’ of which there were 534, of which 93 were in the SMP and 465 in the BDLP, with the proportion of associations being similar (per Fig. 4a) and 24 associations being recovered by both groups. We further minimised this set by removing the set of all associations that were recorded as being seen ‘never’ or ‘rarely.’ In the SMP, there were 23 phenotype associations meeting the criteria, and in the BDLP 79. The SMP contains a slightly higher proportion of this association subset than does the BDLP, with 0.034 and 0.03, respectively. We can also cross-reference this criterion for associations that were reviewed as not being well established, to provide a smaller set of hypothetical associations. These associations, of which there are 23, are shown in Table 4. The majority of 16 associations are concerned with fibromyalgia, with vasculitis, hypertrophic cardiomyopathy (HCM), and asthma also appearing. Of the 23, a majority of 18 were not present in the BDLP.

**Table 4:** The set of social media phenotype associations that were reviewed by a clinician as being ‘Not established but feasible’ but were seen at least ‘sometimes’

Disease	Phenotype	BDLP	Seen in clinic
Asthma (DOID:2841)	Emphysema HP:0002097	True	3—Sometimes
HCM (DOID:11984)	Respiratory insufficiency HP:0002093	True	3—Sometimes
HCM (DOID:11984)	Abnormal blood ion concentration HP:0003111	False	3—Sometimes
HCM (DOID:11984)	Abnormal vertebral morphology HP:0003468	False	3—Sometimes
Vasculitis (DOID:865)	Abnormality of the foot HP:0001760	False	3—Sometimes
Vasculitis DOID:865	Diarrhoea HP:0002014	False	3—Sometimes
Vasculitis (DOID:865)	Abnormality of fluid regulation HP:0011032	False	3—Sometimes
Fibromyalgia (DOID:631)	Urinary incontinence HP:0000020	False	3—Sometimes
Fibromyalgia (DOID:631)	Tinnitus (HP:0000360)	False	3—Sometimes
Fibromyalgia (DOID:631)	Hearing abnormality HP:0000364	False	3—Sometimes
Fibromyalgia (DOID:631)	Abnormal vertebral morphology HP:0000729	False	3—Sometimes
Fibromyalgia (DOID:631)	Oedema HP:0000969	False	3—Sometimes
Fibromyalgia (DOID:631)	Bruising susceptibility HP:0000978	False	3—Sometimes
Fibromyalgia (DOID:631)	Skin rash HP:0000988	False	3—Sometimes
Fibromyalgia (DOID:631)	Gait disturbance HP:0001288	False	3—Sometimes
Fibromyalgia (DOID:631)	Palpitations HP:0001962	False	3—Sometimes
Fibromyalgia (DOID:631)	Nausea HP:0002018	True	3—Sometimes
Fibromyalgia (DOID:631)	Vertigo HP:0002321	True	3 - Sometimes
Fibromyalgia (DOID:631)	Sciatica HP:0011868	False	3—Sometimes
Fibromyalgia (DOID:631)	Night sweats HP:0030166	False	3—Sometimes
Fibromyalgia (DOID:631)	Diminished ability to concentrate HP:0031987	False	4—Often
Fibromyalgia (DOID:631)	Low levels of vitamin D HP:0100512	True	3—Sometimes
Fibromyalgia (DOID:631)	Food allergy HP:0500093	False	3—Sometimes

These represent a small sample set of phenotypes that could potentially be novel, supported initially by the clinician account of their feasibility and prevalence. The “BDLP” column indicates whether this association was also found in the Biomedical Database and Literature Phenotype.

**Fig. 5:**
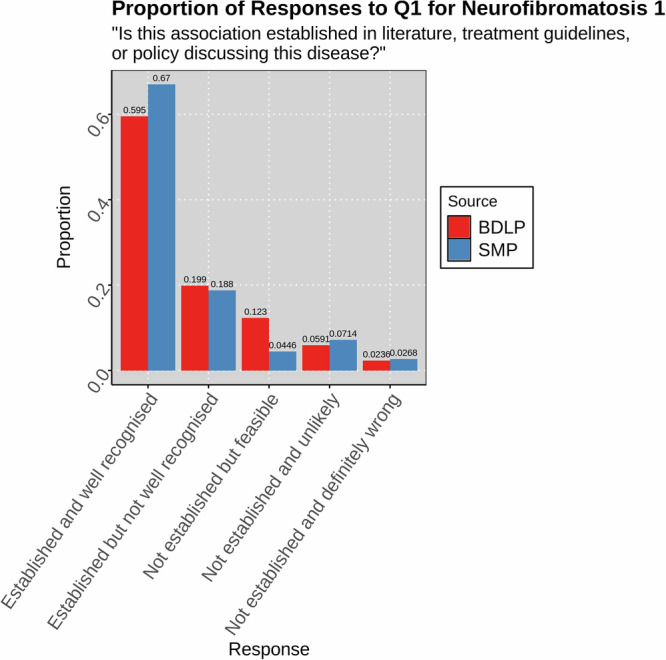
Reviewer-judged validity of associations for SMP and BDLP in Neurofibromatosis 1. Responses were created by a non-clinician expert for Q1 ("Is this association established in the literature, treatment guidelines, or policy discussing this disease?'') for associations with the rare disease Neurofibromatosis 1 in the SMP and BDLP.

Since our investigation focuses primarily on common diseases, we performed a non-clinical evaluation of a rare disease, neurofibromatosis 1. Since this was not based on clinical experience, only Q1 was answered with respect to the literature discussion of the disease. The results, visible in Fig. 5, exhibit a similar distribution to the overall clinical evaluation, with a more obvious trailing tail towards ‘definitely wrong.’

## Discussion

We described the creation of a social media-derived phenotype model for a set of diseases in the form of associations between those diseases and phenotypes. We also collected and combined an associative database from multiple literature analyses and experimental databases, representing the academic perspective on diseases. We linked a set of diseases from these two sets, comparing and contrasting them, with the hypothesis that they would be notably different, reflecting the differences in perspective between the academic community and the public. The results showed that while the social media phenotype semantically recapitulated the academically derived phenotype, the majority of social media-derived associations (64.93%) did not appear in the biomedical database and literature-derived phenotype model, and the vast majority of biomedical and literature-derived phenotypes did not appear in the social media associations (91.18%). Nevertheless, a similar proportion of associations were found to be valid by clinical reviewers. We can, therefore, conclude that the phenotypes derived from social media represent strikingly different perspectives on disease.

The clinical review of a subset of diseases showed that from the clinicians’ perspectives, the social media-derived phenotypes and biomedical and literature-derived phenotypes were similarly valid or well-established. They also showed that for those diseases, the social media-derived phenotypes were seen significantly more often in the course of practice. That the clinical review results showed similar validity, as well as the substantiation of novel associations included subsequently through literature review, indicates that phenotypes novel to the SMP are not generally completely unmentioned in literature. Nevertheless, they did not appear together enough to be considered associated and, therefore, did not appear in the relevant associative dataset. As such, these novel associations may constitute hypotheses or additional evidence for phenotypes that should be considered for further exploration.

In terms of the subset of phenotypes that were not known to clinicians, these can be interpreted in different ways. Where associations are novel to clinicians but appear in literature, it is likely that there is some evidence or association that has not found its way yet into the level of clinical translation that would mean that a clinician knows about them. This is perhaps why there are fewer ‘feasible’ relationships here. In the interests of exploring further the different knowledge and priorities encoded in datasets discussing healthcare entities, similar investigations could be undertaken on routine clinical datasets. These could involve textual analyses, but also analysis supported by a common phenotype profile representational schema^37^. The necessity of exploring the clinical perspective is illuminated by the results of the clinical analysis. While they confirmed that the social media-derived phenotype was similarly aligned to, with a similar distribution of answers across the evaluated diseases, they showed that neither BDLP nor SMP were particularly well-aligned with clinical experience, with more than half of associations in both cases not being seen more than ‘rarely,’ and more than 30% in both cases being considered ‘definitely wrong’ or ‘unlikely.’ We believe that this implies that, in addition to the significantly different perspectives afforded by the BDLP and SMP, the clinical view is yet another majorly different perspective on disease, that diverges significantly from both the scientific and the social media view. These could be mined from the clinical narrative in a similar manner to that described here, as well as integrating structured information such as national reporting statistics or cost coding.

In addition to the evaluation of the social media phenotypes identified in this work, the work is the first to manually evaluate a large number of associations in existing associative databases, that are currently being used in a range of downstream tasks. Outside of alignment with the clinical perspective, the evaluation also identified a large number of phenotype associations marked ‘not established and definitely wrong’ in the pre-existing BDLP set (15.9%). We believe that this necessitates further investigation of the validity of phenotype associations that are derived from text mining of literature. The two studies that we sourced BDLP from validated their text-mined associations by evaluating their recapitulation of expert-curated resources and measuring performance at downstream tasks, though their focus on the identification of novel associations means that these are primarily used to determine cut-offs for associations. This could be of potential importance to the downstream tasks that use these databases, such as differential diagnosis or variant prediction. A more detailed evaluation of phenotype associations could also record demographic information such as years or location of practice or level of seniority, however our requirement that all participants are UK specialists in the reviewed disease with an active practice, provides a high baseline level of experience for reviewers. These factors could also influence the explication of differing intra-context perspectives on disease, as noted above, since questionnaire responses could also form the basis of association development.

Using the results of the clinical review, we were able to illustrate a small set of potentially novel SMP phenotypes by identifying those that were found to be non-established, feasible, and seen in the clinic at least sometimes. The dataset, however, includes a greater number of phenotypes of potential interest, for example those that are seen rarely from the clinician’s perspective may nevertheless be valid. In the subsequent section, we provide a narrative review of those phenotypes and their feasibility for two diseases.

We also provided a non-clinical review of a rare disease, neurofibromatosis 1, to provide evidence for performance on rare diseases (since the clinical review focused primarily on common diseases). In this case, we only evaluated the validity of the association with respect to literature. Though only initial evidence from a single disease, the results show a similar parity of validity between SMP and BDLP. We suspect that this could be because rare diseases and more specific phenotypes could be less likely than common, more general phenotypes, to be mentioned erroneously or in reference to something else. We anticipate that future work that more deeply examines rare diseases could be worthwhile, potentially identifying phenotypes of use in tasks such as disease diagnosis.

Fibromyalgia is characterised by chronic widespread pain alongside a number of non-pain features such as intrusive fatigue, poor refreshment from sleep, poor concentration and short term memory, and hypersensitivity to visual, auditory, and tactile stimuli^38^. Although the exact mechanisms remain unknown, a wealth of evidence shows that altered central nervous system processing can drive or maintain chronic pain in the absence of peripheral nerve or tissue damage^39^. It is possible that some of the sensory non-pain-related symptoms highlighted in the current study, such as tinnitus, palpitations, vertigo and nausea, are also due to central augmentation but have received less attention in the literature. For example, some studies have shown a mismatch between self-reporting of palpitations amongst patients with fibromyalgia, compared to healthy controls, in the absence of any significant differences in objective cardiac measurements^40,41^. Autonomic dysfunction has also been proposed as a potential mechanism for some of the symptoms identified by the SMP, including palpitations and skin manifestations, but objective data to support this theory is currently lacking^40,42,43^. Diminished concentration is a well-established phenotype of fibromyalgia, and was incorrectly marked as ‘not established’ during the validation phase.

Hearing and balance-related symptoms were also identified by the SMP, whereas research in this area is also lacking. Preliminary data suggest a higher handicap relating to the presence of dizziness in patients with fibromyalgia compared to controls^44^. Furthermore, a recent, small-scale cross-sectional study of the impact of tinnitus in fibromyalgia provides preliminary support for the link with central sensation as the severity of tinnitus was associated with the severity of overall symptoms of fibromyalgia and poorer quality of life^45^. Self-reported hearing loss, amongst other sensory symptoms, has also been previously shown to be more prevalent amongst patients with fibromyalgia compared to those with other rheumatic conditions, adjusting for age and sex^46^. Whilst this may represent central augmentation, increased reporting of non-sensory symptoms, including easy bruising, highlights the fact that other mechanisms must be involved.

Some of the unique phenotypes identified by the SMP may reflect the diversity in body systems affected by fibromyalgia, with some symptoms falling beyond the currently acknowledged causal mechanisms. For example, urinary incontinence has been linked to weakened pelvic floor muscles in patients with fibromyalgia, which is, in turn, related to the presence of lower urinary tract symptoms such as urinary incontinence^47^. Although the exact mechanisms are not known, early data suggest impairment of the nerve roots supplying the urinary and anal sphincters^48^. Similarly, a systematic review and meta-analysis have shown that people with fibromyalgia walk with a cycle of shorter length and lower frequency, producing a slower gait^49^. In addition, patients have a higher rate of perceived exertion with the 6 min walking test^50^. Although abnormalities in individual vertebrae morphology have not been shown in the literature, abnormal spinal alignment has been reported^51,52^ and further investigation is warranted.

In contrast to many of the symptoms described above, which generally lack attention in the medical literature, the potential relationship between vitamin D and fibromyalgia has been extensively studied^53,54^. In theory, the mechanisms by which vitamin D may be relevant in fibromyalgia include effects on skeletal muscle, neurotransmitters and neuronal regulation^55^. The issue is that observational and supplemental studies have produced conflicting results. This may be due, at least in part, to the heterogeneity of fibromyalgia and the difficulty in adequately capturing a meaningful change in symptoms at the individual level.

Although there has been much interest in the effect of diet on fibromyalgia, the current evidence base remains inconclusive. A survey of 101 patients with fibromyalgia suggested that the self-reported frequency of food allergy is likely to be higher than the general population^56^. Whilst further evaluation is needed, this is consistent with the observation that patients are more likely to report allergies more generally, and may also represent hypersensitivity rather than true allergy similar to drug hypersensitivity that is seen in fibromyalgia^57^. It also suggests that fibromyalgia patients are seeking out dietary measures, which is understandable given the lack of pharmacological treatments and focus on lifestyle measures more generally^58^.

Although it is difficult to envisage electrolyte abnormalities (synonymous with blood ion concentration abnormality) occurring in unmedicated patients as a direct result of hypertrophic cardiomyopathy, it would be feasible for patients treated for heart failure or in those with concomitant renal disease. The relationship between HCM and electrolyte abnormalities may be overlooked in the scientific literature due to the focus on cardiac-specific biomarkers, such as B-type natriuretic peptide (BNP) and Troponin. Electrolyte abnormalities are related to prognosis in patients with heart failure from any cause^59^. The idea that routine blood tests, which international guidelines recommend are taken at a patient’s initial assessment^60^, could help refine the understanding of disease trajectory and open an immediate avenue for investigation.

Meanwhile, the appearance of respiratory insufficiency as unestablished appears to be a labelling error, as breathlessness due to heart failure is well-described in the scientific literature. Researchers describe most cohorts according to their levels of breathlessness using the New York Heart Association (NYHA) classification.

Abnormal vertebral morphology may be connected to hypertrophic cardiomyopathy through a neuromuscular condition called Freidrich’s Ataxia, where patients are affected by scoliosis and an HCM-like cardiac phenotype^60^. The relationship may be describing affected individuals who have not yet received a formal diagnosis of Freidrich’s Ataxia. A link to HCM itself would be surprising and challenging to explain since changes in this condition are caused by abnormalities of the sarcomere and are confined to the heart.

Using the ontology to contrast the themes and categories of the associations, it was clear that the social media phenotype was heavily skewed toward constitutional symptom phenotypes. Constitutional symptoms are defined in the Human Phenotype Ontology as “*[...] indicating a systemic or general effect of a disease and that may affect the general well-being or status of an individual*”, with further guidance on the classification specifying that the category is defined by phenotypes that affect patient quality of life. The largest contributor to these new associations was, by far, pain, and its more specific subclasses, which was also the largest contributor to overall novel associations, at 6%. Other large contributions from constitutional symptoms came from phenotypes including fatigue, impairment of activities of daily living, night sweats, and indigestion. Upon further investigation of constitutional phenotypes and their accordance with disease areas, we found that there was a greater focus on abdominal, endocrine, and reproductive system disease.

Despite the large number of additional pain-related associations, we showed that most of these were concentrated around more general, less specific, phenotypes. Conversely, the BDLP pain phenotypes were more distributed across the full set of pain phenotypes defined in the Human Phenotype Ontology. We suggest that this is partially resulting from the public not knowing more advanced and technical medical terms for more specific kinds of pain, but also that these more specific terms are not necessarily relevant to the context in which a symptom is being discussed on social media. For example, there were no associations for ‘precordial pain,’ but there were mentions of its more general parent, ‘chest pain.’ In this case, ‘precordial’ is a technical term that many members of the public may not be familiar with, and the use of it in a social media conversation does not necessarily communicate an informative difference, at the cost of wider interpretability. Conversely, the specifying difference between chest and precordial pain may be highly relevant in the context of an academic study or clinical care.

Some relatively simple terms were also not represented in the SMP, however, such as ‘wrist pain,’ and we believe that this points towards our methodology. An area of difficulty in comparing these datasets is our use of a relatively stringent significance requirement for consideration of an association in the analysis. This is closer to the Pilehvar et al.^9^ methodology, which used a false discovery cut-off with a Fisher exact test. Meanwhile, the Kafkas et al.^8^ work provided all associations with positive NPMI scores, reporting that a threshold of 76 phenotypes results in maximal similarity to manually curated associations. Our investigation used a very exclusive false discovery rate of 0.0005 in an effort to yield higher-quality associations for our subsequent analysis, with the aim of discovering novel phenotype associations with high plausibility. Moreover, we did not want to align our significance testing with expert ground truth or recapitulation of biomedical databases, as the other two studies did, since our goal was to identify associations that are not necessarily aligned with this perspective on disease. Nevertheless, our approach yielded a similar distribution of validity among our expert clinical analysis. In using this approach, however, we necessarily exclude many potentially valid associations, and this becomes more likely where concepts become more specific in the knowledge graph, which likely contributes to the concentration of SMP phenotypes on more general phenotypes (seen, for example, in Fig. 3). While this makes it difficult to draw conclusions from differences in the appearance of more specific phenotypes between the two datasets, especially where they do not appear in the more strict SMP, it does not preclude the thematic analyses upon which we have focused in this paper and places a greater interest on associations that were identified. A wider exploration of methods for scoring co-occurrence should be considered as future work, as well as methods for identifying and evaluating interesting associations, even where they fall below a relatively high significance threshold. Ultimately, multi-contextual phenotype models should be developed using equivalent methodologies for a more fair comparison, though we believe the current study provides initial evidence that a social media phenotype model is a valuable resource, worthy of further investigation.

The data were sourced from a wide range of social media sources. These included a majority from generalised social media such as Twitter, but also Reddit, which is organised into many topic-based sub-fora, as well as other sources. Twitter (now X) users tend to over-represent the younger population, especially in the 33–44 age group, and have a higher level of final education and income. USA Twitter users are dominated by the white population and those who tweet more tend to be a small group of female users, according to a survey carried out by the Pew Research Centre^61^. The under-representation of older, poorer and black users is, in principle, likely to show up in fewer complex diseases associated with ageing, such as arthritis, degenerative cardiovascular diseases, deafness and dementia. Similarly, we would expect to see fewer diseases associated with social deprivation and malnutrition^62^. By their frequency in the population, we would expect fewer messages on specific, very rare diseases.

Furthermore, it is highly likely that there are many sub-contexts expressed in these data, for example, differences between discussions of disease informed more by more general social opinions and those informed by the more specific and technical understandings exhibited by those with direct experience of a disease. For example, previous work found that while patients used different language and had different priorities, they knew and used advanced medical terminology in online conversations^29^.

While this initial work shows differences in disease representation across contexts, additional work should be undertaken to identify differences within single contexts. As mentioned above, this could inhere in the exploration of differences in perspectives across different social media websites, and, therefore, the cohorts that use them. Intra-domain diversity may also be explored in the BDLP context, such as whether particular authors or journals exhibit different understandings of diseases—for example, journals more or less specific to a given disease. With the future development of a clinical phenotype, data could be explicated on a number of factors, such as the role or seniority of the person writing the document.

Our investigation chose a strong representation of underfocused diseases to explore, such as fibromyalgia, or many diseases primarily in women’s health, and identifies large modules of potentially novel phenotype associations for those diseases. We anticipate that these should be followed up for more advanced analysis to precipitate a more advanced understanding of those diseases. Intra-domain analysis and stratification could also build upon this initial work in explicating views on these diseases, as well as, for example, gendered experiences or perspectives on disease. Such approaches, however, provide difficulties, since gender and other demographics are not often included with social media datasets.

There were entire facets for which the BDLP is overall more connected than the SMP. For example, it describes a far greater number of thoracic cavity phenotype associations. We suspect that this could relate to the number of layperson synonyms defined in the HPO for those phenotypes since those synonyms contributed to the text-mining vocabulary used by this study. A study describing the development of layperson synonyms in HPO reported 0% coverage for the thoracic category^63^. To a lesser extent, voice phenotypes are also under-represented in the SMP, despite that group being reported as having 44% layperson synonym coverage. This perhaps speaks to the relatively small size of the voice facet of HPO, which is largely concerned with highly technical terms, whose layperson synonyms form complicated compound phrases that are unlikely to be found in the conversational text, e.g., ‘weakness of the vocal cords.’ Other components, such as ‘cries,’ are mostly associated with babies, who are unlikely to be expressing themselves on social media. Facets that are under-expressed in the SMP could represent those patients are less aware of, or less interested in, or they could indicate poorer alignment of the vocabulary with the language they use.

At a more basic level, the inherently error-prone nature of text-mining and large-scale association mining, as well as the shift in language meaning across contexts, mean that extracted disease-phenotype associations may not actually reflect true biomedical relationships, and scepticism should be employed when considering any uncurated associations. The NPMI measure objectively measures co-occurrence in text (affecting both the BDLP and SMP), and is not based on actual incidence, and it is therefore limited in accuracy. For example, the phenotype anorexia (HP:0002039) is defined as “A lack or loss of appetite for food (as a medical condition),” which is distinct from the disease anorexia nervosa (DOID:8689). This distinction may be lost in a public context, where ‘anorexia’ is often used as a referent for the disease, and more rarely for the phenotype of poor appetite. These limitations are, however, a component of any co-occurrence approach to determining relationships between biomedical entities, with scientific literature also referring to the disease with the unqualified ‘anorexia’ in some cases^64^. Further complicating this example is ‘anorexia’ being a substring of ‘anorexia nervosa,’ meaning that in many text mining approaches, all instances of ‘anorexia nervosa’ in the text would also be labelled as an instance of anorexia.

Improvements to the text mining methodology could also mitigate issues with limitations to the use of formal terminologies for text mining. Particularly, the transactions provided by White Swan were determined using keyword matching and, therefore, required exact mentions of labels included in the vocabulary to link an entity. This approach was shared by the Kafkas et al.^8^ approach. State-of-the-art approaches to text mining in a healthcare context often employ contextual embedding similarity to identify and link mentions using labels not explicitly defined in the underlying vocabulary^65^, and the Pilehvar et al.^9^ approach used such a method. The employment of this kind of method would aid in linking mentions that are not pre-defined in the relevant vocabularies, which would be especially beneficial in the use-case of picking up mentions from social media, although these approaches come at the cost of an increased error surface for erroneous annotations and additional complications in determining appropriate cut-offs. In a similar manner to the more strict statistical boundary used in our approach, a keyword approach to NER makes it difficult to infer from the absence of phenotypes from the SMP but does not affect the interpretation of their appearance, especially where those associations do not appear in the literature dataset. More advanced NLP methods could also be used to disambiguate between mentions of diseases and phenotypes that share the same label, for example, by training embeddings that encode different senses of concepts that share the same labels. In our investigation, and in others that rely on keyword-based matching, single mentions may be ascribed to both the phenotype and disease sense of the words.

Neither the SMP developed here nor the previous works that make up the BDLP via literature mining, consider authorship across posts. This could potentially be a source of bias, for instance, that individual authors may make many posts, and therefore have an outsized influence on the representation of a particular disease. We believe that this effect is likely small, though could make for an interesting follow-up study, potentially exploring other splitting factors such as demographics, geographic locations, or journals. In a potential clinical data-derived phenotype, factors such as role and seniority could be considered.

For these reasons, ultimately, while our work identifies a large number of hypothetical relationships between diseases and phenotypes that are not reflected in current academic databases, further work must be done to explore them and to identify what, if any, scientific or clinical utility they have. This limitation is also relevant to the associations recovered by the other studies we explored that make up the BDLP, and we anticipate a programme of research that surrounds the alignment, comparison, and evaluation of multi-contextual disease phenotypes in a single methodological context. Future work could explore particular associations, following up to identify additional evidence and explanations, correlating with other types of data or performing causative analysis to identify and eliminate confounding factors. Meanwhile, the associations could also be explored in the context of their contribution to downstream tasks such as differential diagnosis or causative variant prediction. Future work could also include more direct alignment and extraction of associations to other medical vocabularies and ontologies, such as MONARCH, which could provide benefits to analysis through the integration of data already contained in those ecosystems^66^.

One previous study has also identified a critical need for correlating digital phenotyping data with epidemiological data^32^. Recent efforts such as BioLink^67^ aim to formalise and harmonise biomedical entity associations, however they do not include extensive vocabularies for text mining, and do not include a rich metadata language for describing the derivation and provenance of calculated associations. In our review, while clinicians were largely able to categorise all phenotype associations into a small number of categories, with a relatively small number percentage of associations being marked as ‘other’ or ‘unknown,’ this required a lot of manual work, and the automated inference of the nature of these relationships from textual context could be considered a task for future work. As a secondary output of this work, we consider that the type of association judgements by clinicians could form an initial gold standard by which such a method could be evaluated.

We also envision that these hypothetical relationships can be used as prompts for patient interaction and involvement, building an integrated evidence base for introducing changes to clinical practice that more closely reflect and serve public and patient priorities. Hypotheses can be evaluated and correlated with other sources of patient voice data, including patient-reported outcome measures, and these processes can also be used to query the exact nature of the relationships and perspectives being explored, ensuring that they are more fully understood, and employment of these methods could also help to control for bias in social media demographics. We anticipate that the use of deep phenotyping data from a range of multi-contextual resources can be employed as a contributing device in an increasing drive toward patient-centred research and care.

In conclusion, we developed a social media-derived phenotype model of disease to represent public and patient perspectives on the disease and its signs and symptoms. We have demonstrated that this phenotype model expresses a significantly different perspective than that expressed by biomedical databases and literature. Moreover, we identified a large number of novel associations that were not represented in the biomedical and literature model. We anticipate that this knowledge resource can contribute to an improved understanding of human diseasome across healthcare research and implementation and that analysis of diverse data sources can contribute to a fairer and increasingly patient-centred approach to medicine.

## Methods

The methods used to produce our results are available online at https://github.com/reality/sd_paper. This project was reviewed for research ethics by the Science, Technology, Engineering and Mathematics Committee at the University of Birmingham, and granted full ethical approval with identifier ERN_2022-0241 and ERN_0241-Jun2023 amendment. The board determined that informed consent was not necessary because this was covered by the use agreements of the websites, and White Swan Charity’s agreements with the companies that provided the data.

### Dataset

We obtained a database of transaction data describing concurrent mentions of diseases and phenotypes in social media posts from the White Swan charity. Each entry in the transaction set refers to a single post made by a user. White Swan is a registered charity in England and Wales (1176486), that aims to improve health and well-being through artificial intelligence technology and analytics. White Swan purchased social media posts mentioning a list of disease keywords via Twitter (now known as X) and Socialgist. Diseases were selected according to the priorities of the White Swan charity, and purchased keywords were determined for those diseases using DO labels as a basis and improved through manual curation. Socialgist is a company that provides access to social media data and has been used in a range of disease analyses described in the literature. Twitter posts were accessed from Twitter, while all other posts were obtained via Socialgist. The list of purchase keywords is available via the repository noted above. Posts with equivalent text content were removed. We did not use any strategy to control for uniqueness or non-uniqueness of post-authors, in order to match methods used for literature mining, which does not consider representation of authors across articles. Those posts were analysed using a keyword matching approach, identifying mentions of Human Phenotype Ontology (HPO) and Disease Ontology (DO) classes, using vocabulary identified for those phenotypes and diseases across identifiers across biomedical ontologies, using the method described in Slater et al.^68^. DO mentions in the transactional dataset were limited to those for which keywords were explicitly defined, to control for incomplete representation of diseases which were not intended to be received. The dataset covers a period from 1 November 2019 to 1 November 2021.

### Social media phenotype (SMP)

We propagated mentions of classes for phenotypes across the set of transaction records to superclasses. For example, any mentions of ‘low back pain’ would also be considered mentions of ‘back pain,’ and ‘pain.’ As described by Kafkas et al.^8^, this was achieved using a modified measure of Normalised Pointwise Mutual Information (NPMI) that takes into account subsumptive hierarchy, shown in Eq. (1). NPMI is an objective measure of co-occurrence of items in a database. In this case, the items are HPO and DOID classes. These mentions are seeded by explicit mentions and expanded using the true path rule of subsumption according to the underlying ontology (for example, all mentions of low back pain are also mentions of back pain). In the given equation, *C* and *D* refer to a specified pair of classes, and *n* identifies number of occurrences in the database of those class, with *n*_*C*,*D*_ defining the number of documents in which those classes co-occur. The intuition of the equation is that classes that appear together more frequently with respect to the total number of documents in the database are assigned a greater score. Subsumption was identified using the ELK ontology reasoner^69^. We did not propagate disease mentions due to the limited number of diseases being examined, causing potential bias to arise due to incomplete composition from subclasses.

1 $${\rm {npmi}}(C,D)=\frac{{\rm {log}}\frac{{n}_{C,D}\cdot {n}_{{\rm {tot}}}}{{n}_{c}\cdot {n}_{D}}}{-{\rm {log}}\frac{{n}_{C,D}}{{n}_{{\rm {tot}}}}}$$

We calculated NPMI values for every combination of disease and phenotype. We then used a Monte Carlo bootstrapping approach to simulate a randomised transaction set with the same size and shape 2000 times, using NPMI values calculated from those simulations to identify p-values for every association. We confirmed the normality of scores across the simulations visually.

We excluded from further consideration any associations with a non-positive NPMI value, since this study is concerned with positive associations. We also excluded diseases and phenotypes that appeared in fewer than 0.01% of transactions. We did this to avoid skewed NPMI values for associations involving classes that appeared very infrequently. We also removed highly co-linear diseases and phenotypes, with a cut-off of 0.75 NPMI. This was to control for the definition of diseases and phenotypes in DO and HP with shared labels, referring to the phenomenon in the context of a disease and a phenotype, respectively. The remaining set of associations comprises the SMP. We then calculated *q*-values^70^ for all SMP associations, using an acceptable false discovery rate of 0.0005, with associations meeting this threshold being considered significant in the context of this study. We selected this high statistical threshold for significance to partially control for the inherent noisiness of social media text data. We also performed a review of phenotypes included in associations to identify any with associated labels that would be unlikely in a public context to imply a similar phenomenon to the actual phenotype (e.g. plethora (HP:0001050)), or that had erroneously associated labels (no social interaction (HP:0008763) also had label ‘social interaction’). These phenotypes were discluded from the final set of associations and, therefore, removed from further consideration in our experiment. The combination of NPMI and *q*-values is intended to identify disease-phenotype pairs that are over-expressed with respect to their appearing together in the text corpus.

### Biomedical Database and Literature Phenotype (BDLP)

To construct a resource representative of existing background knowledge resources describing biomedical databases and literature, we collected phenotypes from several resources: the set of text-mined literature associations from Kafkas et al.^8^, semi-automatic disease-phenotype associations from Kafkas et al.^8^, and the text-mined literature derived phenotypes from Pilehvar et al.^8,9^. All of these datasets use HPO to describe phenotypes. However, Kafkas et al.^8^ used ICD-10 for diseases, while Pilehvar et al.^9^ used MONDO. We linked diseases to DO using the database cross-references defined in MONDO and DO. For unlinked diseases, we developed manual mappings, where possible, and contributed these associations to DO. There remained a number of DO disease concepts that we were not able to identify or develop mappings for: swine influenza (DOID:0050211), Human cytalomegalovirus infection (DOID:0080827), Gigantism (DOID:2446), Phimosis (DOID:2712), renal carcinoma (DOID:4451), Kyphosis (DOID:4667), and Hordeolum (DOID:9909). These diseases are available in the subsequent SMP dataset but are not considered in the differential analysis between BDLP and SMP. We consolidated phenotype associations from the three sources listed above for all diseases with a cross-reference to DO using a union approach, in order to produce a maximal set of phenotype associations derived from literature and curated resources for each disease. These consolidated associations made up the Biomedical Database and Literature Phenotype (BDLP) dataset.

### Identifying novel associations

So as to identify any associations recovered from the social media text, that were not contained in the BDLP, we defined a subset of the total set of social media-derived associations for each disease. The subset ‘novel’ contains all phenotype associations for which there is no equivalent or more specific association in the BDLP. We further label as laconic the SMP phenotypes that do not have a more specific significant association. In this way, we can identify subsets of social media-derived associations for each disease that are significant, maximally specific, and distinct from those in the literature-derived set. We developed a website at http://phenotype.digital/for exploring and comparing the phenotypes of diseases across the two contexts. ChatGPT was used to aid in the development of the website front-end, which was implemented in NodeJS. The web interface also provides an indication of whether associations are included in the HPO annotations database. We used the 2024-04-19 version of the HPOA annotations. We should note that this is different from the version of HPOA described by Kafkas et al.^8^ who used a cut-off of 2020-10-12.

### Clinical review

We sought clinical collaborators to review associations. All reviewers are consultant clinicians with an active practice in the disease(s) they reviewed in the UK. Reviewers were blinded to the source of the associations, and their order was randomised. Reviewers were not required to review all associations for a given disease. The questions asked were:

1. Is this association established in the literature, treatment guidelines, or policy discussing this disease? (established and well recognised, established but not well recognised, not established but feasible, not established and unlikely, not established and definitely wrong).
2. What kind of association is this? (symptom or sign, comorbidity, complication or can be sequel of, side-effect of treatment, other associated phenotype, unknown).
3. How often do you recognise this association in the course of your clinical practice for this disease? (1—never, 2—rarely, 3—sometimes, 4—often, 5—very often).

Frequencies were accorded as a Likert scale^71^. Guidance was given on the interpretation of the Likert scale in this case: “The question concerning how often you recognise an association in your clinical practice refers to how often you recognise or consider the possibility of the phenotype when seeing a patient with the condition above. It should not be influenced by how often you encounter the disease or possible cases of the disease in the course of your practice. For example, only use ‘rarely’ when you rarely see patients with that phenotype in that disease cohort, not when you rarely see patients with that condition." The wording of the questionnaires was determined by a co-author and clinical reviewer (W.B.). Specific definitions for words such as ‘feasible’ were not given, to facilitate clinical perspective interpretation of the associations. Guidance on definitions were given for ‘phenotype’ and ‘association’ however: “A phenotype is an observable trait in an organism - in this case, humans.” and “An association is a link between a disease and a phenotype.” Reviewers were instructed on answers that would only be relevant for a sub-type of the disease: “If the association is only valid for a sub-type of the disease (e.g. only in a genetic aetiology, but not in sporadic cases), please answer as you would for the relevant disease sub-type.” The reviews were conducted via a web application implemented into the digital phenotype website, and which was developed to permit additional review and collection of expertise in the future. The diseases reviewed were bronchiectasis (DOID:9563), pancreatic carcinoma (DOID:4905), systemic lupus erythematosus (DOID:9074), skin carcinoma (DOID:3451), dermatitis (DOID:2723), asthma (DOID:2841), hypertrophic cardiomyopathy (DOID:11984), pulmonary embolism (DOID:9477), vasculitis (DOID:865), congestive heart failure (DOID:6000), chronic obstructive pulmonary disease (DOID:3083), and fibromyalgia (DOID:631). Non-clinical evaluation of phenotype associations for neurofibromatosis 1 (DOID:0111253) was performed by author PNS on Q1 only, with reference to literature rather than clinical experience.

### Evaluation and analysis

To measure how well the SMP semantically recapitulated the BDLP, we calculated an area under the curve (AUC) by ranking semantic similarity scores calculated using the Resnik method^72^, comparing the phenotype profiles of each pairwise combination of matched diseases in the BDLP and SMP. Semantic similarity was calculated using the Semantic Measures Library^73^. We used the Klarigi tool to perform compositional analysis of phenotypes^74^, automatically correcting inclusion scores for subsumptive phenotype relationships implied by the underlying ontology. A combination of Groovy and R were used to perform the valuation. Figures were produced using the ggplot library.

### Reporting summary

Further information on research design is available in the Nature Research Reporting Summary linked to this article.

## Supplementary information

Editorial Policy

Reporting Summary

## Data Availability

The result data and intermediate data are available via https://github.com/reality/sd_paperand https://phenotype.digital/. The derived associations for the BDLP and SMP are available from a data repository^36^. Raw transaction data is not made available as its ownership is retained by White Swan charity.

## Code availability

The code for experiments described in this paper is available from https://github.com/reality/sd_paper.
